# Mechanisms Underpinning Dynamic Impact Resistance Reinforcement by Multi-Scale Synergistic Effect Through Nano-Silica and Carbon Hollow Microsphere

**DOI:** 10.3390/polym17192592

**Published:** 2025-09-25

**Authors:** Yingying Yu, Cheng Yang, Yaxi Zhang, Linjia Wang, Hong Wang, Fandong Meng, Fanyi Meng, Tao Wang, Zhenmin Luo

**Affiliations:** College of Safety Science and Engineering, Xi’an University of Science and Technology, Xi’an 710054, China; yyyu2022@xust.edu.cn (Y.Y.); cyangxust2024@163.com (C.Y.); komoriyui_1152@outlook.com (Y.Z.); wanglinjia@stu.xust.edu.cn (L.W.); 17308387551@163.com (H.W.); mengf@xust.edu.cn (F.M.); christfer@xust.edu.cn (T.W.)

**Keywords:** dynamic energy absorption, flexible impact protection, carbon hollow microspheres, nano-silica, multi-scale synergistic effect

## Abstract

Synergistic improvements in lightweight design, flexibility, and energy absorption efficiency are a long-standing issue of flexible impact protection materials. This study proposed a multi-scale synergistic enhancement method, where nano-scale nano-silica (NS) and micro-scale carbon hollow microspheres (CHMs) were adopted to enhance the dynamic impact property of the flexible polydimethylsiloxane (PDMS). The mechanical behavior of the as-prepared composites under a broad range of strain rates (10^−3^–4500 s^−1^) was systematically investigated. For the composite with 1 wt.% NS and 3 wt.% CHM, the compressive strength under quasi-static conditions reached 39.73 MPa, representing a 67% improvement over the pure PDMS. Under dynamic impact (4500 s^−1^ strain rate), the force transmits through the NS nanonet with the energy absorbed through crushing of the CHM with a hollow structure, which elevates the specific energy absorption properties to 40.18 J/g (for the composite with 1 wt.% NS and 3 wt.% CHM), which owns a 179% enhancement compared to the composite with only 4 wt.% CHM. This work established a novel method for a hierarchical “rigid-flexible energy absorption” structural design of lightweight impact-resistant materials.

## 1. Introduction

Chemical manufacturing involves flammable and explosive chemicals, high-temperature and high-pressure reactions, and highly concentrated energy [1,2,3,4], posing a great threat to the safety and health of operators. Thus, it is of great importance to improve the comprehensive performance of impact protection materials. In general, impact protection materials are required to exhibit low density to ensure wearability and flexibility, high-efficiency energy dissipation capability to withstand explosive impact loads, and excellent chemical stability to resist corrosive media. However, mainstream impact protection materials face severe limitations: Metal–matrix composites (e.g., titanium alloy/boron carbide ceramic systems) [5,6,7,8] offer high strength but typically have densities exceeding 2.8 g/cm^3^, resulting in excessively heavy protective equipment that severely reduces the efficiency of operators. Polymer materials [9,10,11] are lightweight but suffer from low strain-rate sensitivity, resulting in a 40% reduction in energy absorption efficiency under dynamic load compared to that under quasi-static conditions. Among the typical polymer materials, polydimethylsiloxane (PDMS), with ultra-low density, broad temperature range elasticity, and exceptional chemical resistance [12,13,14], serves as an ideal matrix for impact protection materials. However, its intrinsic mechanical properties are insufficient: Pure PDMS exhibits low compressive strength, barely providing effective retardation of high-speed fragment penetration. The energy absorption density of pure PDMS is only 2.5 J/g, far below the critical threshold for chemical safety protection [15,16,17]. As a result, reinforcing components were frequently introduced to optimize the mechanical properties of the polymer materials.

Carbon hollow microspheres (CHMs), as a lightweight reinforcing component, can theoretically achieve high energy absorption density through shell buckling collapse and cavity compression mechanisms. Previous studies have investigated the effect of CHM content on the mechanical properties of CHM/PDMS composites [18]. It is demonstrated that proper CHM content can improve the interaction force between CHM and PDMS by forming a dense stacking structure, leading to a 124.68% increase in compressive strength under quasi-static load and an increase of over 1200% in energy absorption under dynamic impact. Nevertheless, several issues still remain in CHM/PDMS composites due to the modulus mismatch between rigid CHM and soft PDMS, significantly reducing the effective stress transfer efficiency under high-strain-rate impact, which causes a serious decay in energy absorption density.

Compared to traditional micron-sized fillers, nanoparticles demonstrate significant advantages in enhancing polymer mechanical strength and thermal stability due to their unique nano-scale effects. Among them, nano-scale silica (NS) has emerged as a research hotspot in composite materials due to its distinctive physicochemical properties [19,20,21]. NS particles typically range from 1 to 100 nm in size, possessing extremely high specific surface area and surface activity, which underpin their significant application value across multiple fields. This surface activity stems not only from ubiquitous van der Waals forces but also, particularly in PDMS matrices, from special interfacial interactions with siloxane segments, providing a microscopic basis for material performance tuning. Séverine et al. [22] prepared nanohybrid hydrogels via the crosslinking polymerization of N,N-dimethylacrylamide (PDMA) in a dispersion of silica nanoparticles. The introduction of silica nanoparticles significantly improved the mechanical properties of the hydrogels, concurrently increasing both stiffness and nominal strain at failure. Ahmed et al. [23] investigated the effects of different ratios of multi-walled carbon nanotubes (MWCNTs) and nano-silica (SiO_2_) on the mechanical and electrical properties of glass fiber-reinforced epoxy (GRE) composites. Nano-silica particles can be effectively filled into the molecular gaps and micropores of the resin matrix to improve the density and rigidity of the matrix, which could enhance the mechanical strength of the polymer-based composite. Jayabalakrishnan et al. [24] systematically explored the synergistic effects of acrylonitrile butadiene rubber (ABR), NS, and e-glass fiber within an epoxy resin system. They revealed that incorporating NS not only systematically enhanced the tensile modulus and flexural strength of the composites but also significantly improved their impact resistance, with fracture toughness indicators and energy dissipation efficiency demonstrating breakthrough increases. Rajasekaran et al. [25] experimentally studied the effect of NS particle dispersion in luffa fiber-reinforced epoxy composites on their mechanical and thermal properties. They showed that the incorporation of nano-SiO_2_ particles significantly improved both the mechanical and thermal properties of the composites. At 1.5% nano-SiO_2_ dispersion in the epoxy resin, the flexural strength, impact strength, and thermal conductivity of the composite increased by 157.58%, 66.9%, and 47.53%, respectively. Overall, benefiting from the high specific surface area and reactive surface silanol groups, NS particles can effectively fill interfacial micro-gaps and enhance interfacial bonding strength through Si-O-Si covalent bonding, which can contribute to extending the fracture path and augment the energy dissipation of the as-prepared composites [25].

In the presented study, a novel method was proposed to optimize the mechanical performance of the PDMS, especially for the dynamic impact resistance, through a multi-scale synergistic effect. Nano-scale nano-silica and micro-scale carbon hollow microsphere were adopted as the reinforcement components. The mechanical performances of the composite under quasi-static and high-speed conditions were studied systematically through tailoring the proportion of the reinforcement components introduced in the polymer matrix. In addition, the thermal stability of the as-produced composites was investigated to explore the possibility of the NS-CHM/PDMS composite for multifunctional protection.

## 2. Experimental Section

### 2.1. Preparation of CHM and NS-CHM/PDMS Composite

The PDMS matrix (grade BD6184) was obtained from GuiNie Advanced Materials Co., Ltd. (Hangzhou, China). The precursor of the CHMs, phenoset microspheres (BJO-0930), was supplied by Maroya New Material Co., Ltd. (Shanghai, China), with their basic properties detailed in Table 1. The NS (purity ≥ 99.5%, average particle size 15 nm) was obtained from Aladdin Biochemical Technology Co., Ltd. (Shanghai, China).

The preparation of the CHMs used in this study was described in detail in our previous research [18]. In addition, the vacuum-assisted mixing-casting method was introduced to prepare NS-CHM/PDMS composites. The as-received NS was dried in the vacuum oven at 130 °C for 24 h prior to the composite fabrication process to remove moisture. As reported in our previous research, when CHMs were solely incorporated to enhance the impact resistance of the composite, the optimal concentration was 4 wt.%, where CHMs achieved the most efficient structural arrangement. The close packing effect facilitated a strong interfacial interaction among the CHMs and PDMS, and minimized the excessive interfacial stress at the same time. To minimize the impact of the filler content on the properties of the composites, the overall filler content in this work was fixed at 4 wt.%. To investigate the effects of NS content on the energy absorption performance of the composites, the weight ratio between NS and CHM was tailored, and the detailed ratio and corresponding sample denotations are listed in Table 2.

### 2.2. Characterization

The quasi-static mechanical performances of the NS-CHM/PDMS composites were studied through quasi-static compression tests at room temperature using an Instron 3365 universal testing system. Cylindrical specimens with a diameter of 10 mm and a height of 7 mm were designed, and the crosshead displacement speed was set to 1 mm/min. For each test group, three replicate tests were conducted in displacement-controlled mode. Dynamic impact tests were performed using a split Hopkinson pressure bar (SHPB) system. These voltage signals were converted into stress–strain curves based on the one-dimensional stress wave theory. Dynamic impact tests were carried out at strain rates of approximately 2500 s^−1^, 3500 s^−1^, and 4500 s^−1^, with each test group repeated at least five times. Cylindrical specimens with a diameter of 6 mm and a height of 2 mm were prepared for high-strain-rate testing. Elemental analysis and morphological observation of the samples were conducted using a scanning electron microscope (Quanta 450, FEI, USA) equipped with energy-dispersive X-ray spectroscopy (EDS, IE 250X-Max 50, Oxford Instruments, UK). In addition, the thermal degradation behavior was examined with TG (TG 209 F1, Netzsch, Germany) under a nitrogen atmosphere with a heating rate of 10 °C/min from 25 °C to 900 °C.

## 3. Results and Discussion

### 3.1. Quasi-Static Response: Low Strain Rate

**Figure 1:**
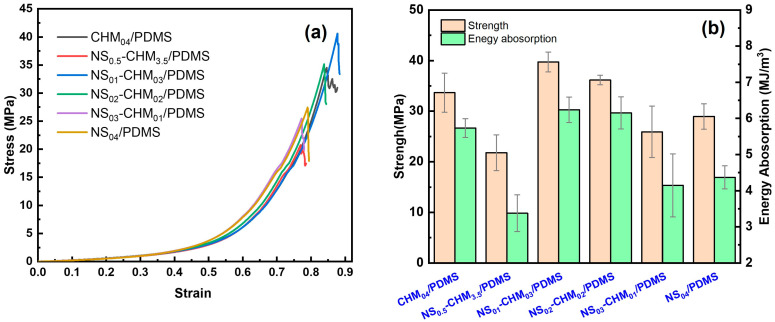
Quasi-static mechanical properties of NS-CHM/PDMS composites: (**a**) representative stress–strain curves; (**b**) strength and the energy absorption performance.

Figure 1a presents the quasi-static stress–strain curves of the NS-CHM/PDMS composites. In the initial stage, the stress–strain curves of all composites exhibit a linear relationship, indicating that elastic deformation occurs at the initial stage, which can be attributed to the elastic deformation of the NS-reinforced PDMS matrix. Then, as the strain further increases into the plastic deformation stage, the curves become nonlinear. In this period, the CHMs are adopted to bear the force, together with the NS-reinforced PDMS matrix. When the strain continuously increases and the materials reach their bearing capacity limit, the stress exhibits a sudden drop, which corresponds to the mechanical failure of the composites, as can be seen from the curves. Significant difference exists among the composites with varying NS-to-CHM ratios: the NS_01_-CHM_03_/PDMS exhibits excellent compressive mechanical performance, indicating its superior structural homogeneity and the greatest load-bearing capacity among the materials tested. The improvement of quasi-static mechanical properties can be attributed to the synergistic effect between NS and CHM within the PDMS matrix, effectively enhancing material strength and toughness. In contrast, the curve for NS_03_-CHM_01_/PDMS exhibits the steepest slope, showing that the composite exhibited high stiffness. However, its bearing capacity drops abruptly at a strain of 0.75, resulting in a decrease in the energy absorption performance. The relatively low compressive strength of composites with high NS content may result from the stress concentration induced by the agglomeration of nanoparticles at high NS concentration without a dispersing agent adopted for anti-agglomeration.

Analysis of the stress–strain relationship further confirms that the introduction of NS significantly affects the mechanical properties, as shown in Figure 1b. It can be seen that the introduction of a small amount of NS has a negative effect on the static compressive mechanical property, compared to CHM_04_/PDMS, where NS is absent. Note that the sum of the filler weight percentages of NS and CHM is kept as 4 wt.%. With respect to CHM_04_/PDMS, introducing 0.5 wt.% of NS leads to the CHM content dropping to 3.5%, which deviates from the optimal CHM content (4 wt.%) according to our previous study [18]. In addition, the NS particles embedded in the PDMS matrix may act as defects, where microcracks may originate, and the mechanical properties deteriorate. On the other hand, the positive effect of NS particles is that they can enhance the stiffness of the PDMS matrix, which decreases the mismatch between the PDMS matrix and CHM fillers during loading, and the NS can also impede the microcrack propagation. In case of a small NS content, the positive effect of NS on the PDMS matrix cannot compensate for the mechanical property degradation caused by the CHM content decrease.

For the composites with two kinds of fillers, the strength of the as-prepared composites initially increases by 17.92 MPa with low NS content (from 0.5 wt.% to 1 wt.%), but decreases by 13.81 MPa as the NS content further increases (from 1 wt.% to 4 wt.%). The optimal compressive strength and energy absorption performance are found in the composite with 1 wt.% NS, which are 39.73 MPa and 6.24 MJ/m^3^, respectively. The optimal strength and the energy absorption performance of NS_01_-CHM_03_/PDMS (which is the optimal composition in the present study) are 118% and 110% of those of the CHM_04_/PDMS (which is the optimal composition in the previous study [18]). Meanwhile, the strength and the energy absorption performance of the NS_01_-CHM_03_/PDMS are 66% and 81% higher than those of pure PDMS. The strengthening mechanism could be explained as follows: Homogeneously dispersed NS particles effectively share the load, hinder the crack propagation, and alter the path of the crack. Meanwhile, the surface of NS owns silicon hydroxyl (-SiOH), which can form hydrogen bonds or covalent bonds with the silicon oxygen chain of PDMS, which can also contribute to the efficient stress transfer. Simultaneously, the localized crushing behavior of the CHMs during the plastic deformation could dissipate the mechanical energy, collectively delaying the overall material failure process [26,27,28]. When the NS-to-CHM ratio is suitable (e.g., 1:3, 2:2) and dispersion is homogeneous, the stress of the as-prepared composite increases gradually during the loading process, and exhibits good deformation controllability. Conversely, improper ratio or filler agglomeration will lead to an obvious decrease in strength and energy absorption performance.

**Figure 2:**
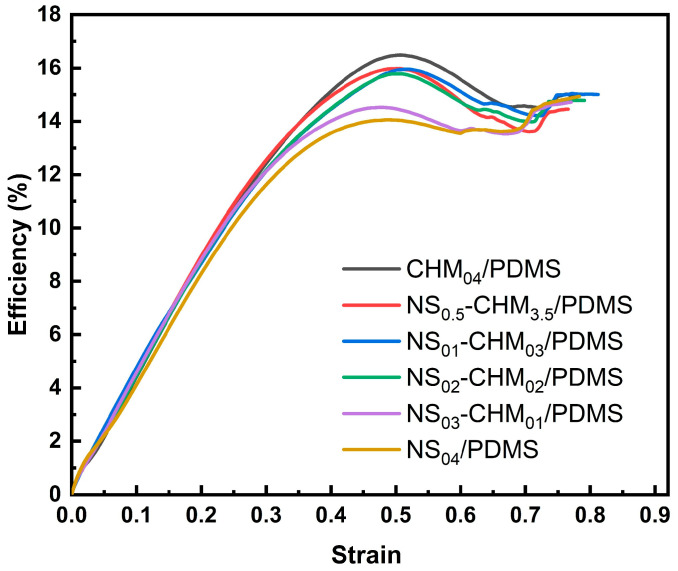
Energy absorption efficiency curves of NS-CHM/PDMS composites.

Figure 2 shows the energy absorption efficiency curves for NS-CHM/PDMS composites with different NS-to-CHM ratios. The efficiency is calculated as *E_A_*/*σ*, where *E_A_* represents the integrated area of the stress–strain curve before a certain point, and *σ* represents the corresponding stress value at this point. All curves show the same tendency with increasing strain of an initial obvious increase, followed by a slight decrease. During the initial compression stage, the structural units that can be used for energy dissipation are gradually activated, resulting in increased efficiency. Then, at high strains, localized failure leads to a decrease in energy absorption efficiency. Among the NS-CHM/PDMS composites, CHM_04_/PDMS demonstrates the highest energy absorption efficiency (approximately 16.37%). It can be primarily attributed to the efficient energy absorption mechanism of the CHM hollow microsphere structure, which absorbs energy effectively through shell deformation or cavity collapse during compression. In contrast, NS, as a rigid filler, mainly transfers the stress through the synergetic effect of the elastic PDMS matrix, exhibiting relatively weaker energy dissipation capacity. As NS content increases and CHM content decreases, the efficient energy absorption mechanism driven by the hollow structure diminishes, and the rigidity enhancement provided by NS is insufficient to offset the reduction in energy dissipation capacity. Although the synergistic effect between CHM and NS contributed to enhancing the energy absorption, it can be inferred that CHM is the dominant contributor to the energy absorption capacity.

**Figure 3:**
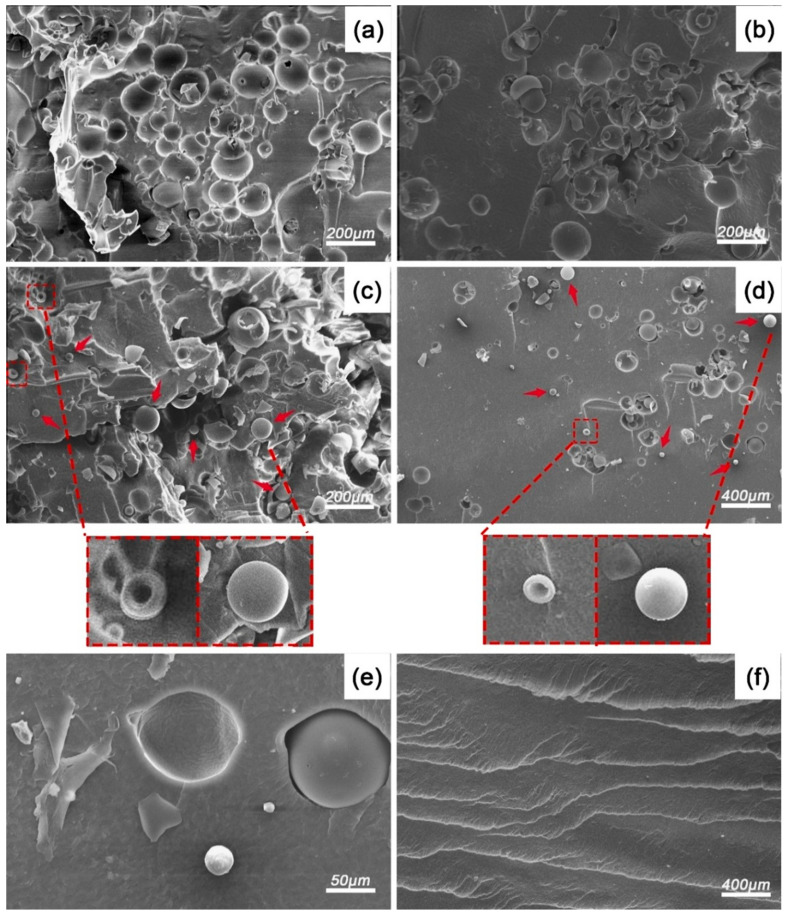
SEM images of the NS-CHM/PDMS composites after quasi-static loading: (**a**) CHM_04_/PDMS; (**b**) NS_0.5_-CHM_3.5_/PDMS; (**c**) NS_01_-CHM_03_/PDMS; (**d**) NS_02_-CHM_02_/PDMS; (**e**) NS_03_-CHM_01_/PDMS; and (**f**) NS_04_/PDMS. The red arrows pointed to the CHMs located in the PDMS matrix.

To further understand the failure mechanism of the NS-CHM-reinforced PDMS composites, the fracture morphology of the as-prepared composites after quasi-static compression loading was studied, as illustrated in Figure 3. As observed in Figure 3a, the fracture surface of the CHM_04_/PDMS composite exhibits numerous regularly arranged pit structures (~50–80 μm in diameter) without intact CHMs, indicating weak interfacial interaction between CHM and the PDMS matrix in the absence of NS, verifying the modulus mismatch of the CHM and PDMS. The residual PDMS tearing marks at the pit edges confirmed that the interfacial debonding can be one of the primary failure mechanisms. In contrast, NS_01_-CHM_03_/PDMS (Figure 3c) displays abundant structurally intact CHMs in the PDMS matrix, demonstrating that the introduction of the NS rigid network effectively works for stress dissipation and shares the load of the CHM to bear.

Notably, deformation can be found on the surface of the CHMs, revealing their participation in energy dissipation, further validating the synergistic effect for the two fillers in improving energy absorption performance of the as-prepared composites. For the NS_02_-CHM_02_/PDMS composite, fewer intact CHMs can be found, and fragments of the CHMs can be seen more frequently. It can be attributed to more efficient stress transfer caused by the increase in the NS rigid network, aggravating the breakage of CHMs. For NS_04_/PDMS (Figure 3f), wave-like fold structures can be seen on the surface of the sample, which demonstrates that the main energy dissipation mechanism is the plastic deformation of the matrix. The above observation demonstrates that a suitable NS-to-CHM ratio could help the composite dissipate energy synergistically through the NS network at the nano-scale and CHM deformation at the micro-scale.

### 3.2. Dynamic Response: High Strain Rate

**Figure 4:**
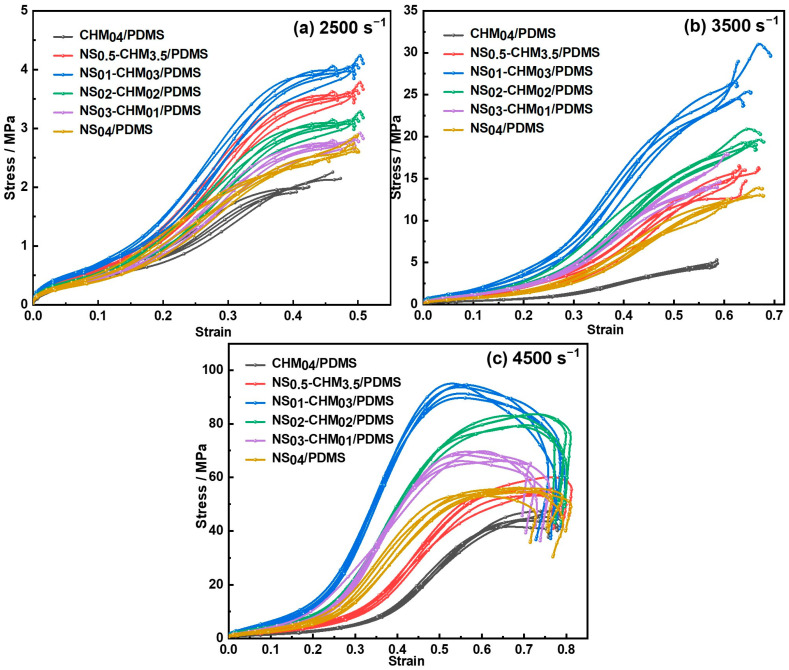
Stress–strain curves of NS-CHM/PDMS composites under impact at strain rates of (**a**) 2500 s^−1^; (**b**) 3500 s^−1^; (**c**) 4500 s^−1^.

**Figure 5:**
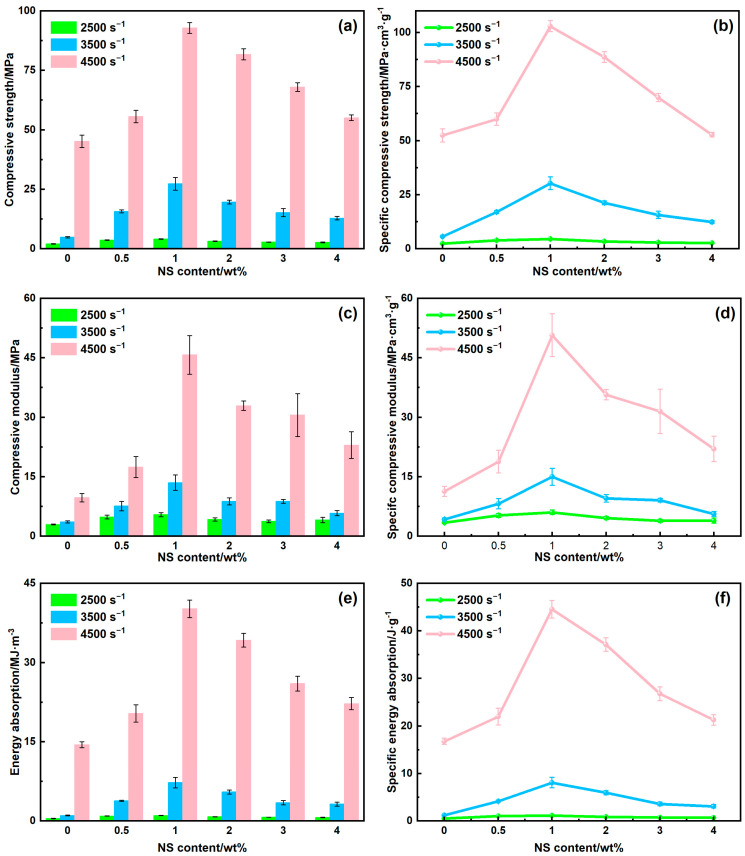
Dynamic impact performance of the NS-CHM/PDMS composites: (**a**) compressive strength; (**b**) specific compressive strength; (**c**) compressive modulus; (**d**) specific compressive modulus; (**e**) energy absorption per unit area; (**f**) energy absorption density.

Figure 4 displays the engineering stress–strain relationships of NS-CHM/PDMS composites under high-speed impact (strain rate of 2500–4500 s^−1^), and the calculated dynamic impact performance of the NS-CHM/PDMS composites, including the compressive modulus, compressive strength, and energy absorption, is illustrated in Figure 5. The curves for CHM_04_/PDMS composites, NS_0.5_-CHM_3.5_/PDMS composites, NS_01_-CHM_03_/PDMS composites, NS_02_-CHM_02_/PDMS composites, NS_03_-CHM_01_/PDMS composites, and NS_04_/PDMS composites were displayed with black, red, blue, green, purple, and brown, respectively. The obtained results reveal that the dynamic impact performance of the NS-CHM/PDMS composites is highly sensitive to the NS-to-CHM ratio and exhibits a consistent trend across the strain-rate range of 2500–4500 s^−1^.

For the composite with two kinds of fillers, the compressive strength of the composite initially increases and then decreases with increasing NS content, exhibiting the same trend as observed under quasi-static conditions. The difference is that, compared to the composite without NS (i.e., CHM_04_/PDMS), a small amount of NS addition (i.e., NS_0.5_-CHM_3.5_/PDMS) can lead to enhancement of the dynamic mechanical property, while it deteriorates the quasi-static mechanical property. This difference indicates that, for high-speed impact, the modulus mismatch between CHM and the matrix plays a dominant role in the dynamic mechanical failure. At 1 wt.% NS loading (NS_01_-CHM_03_/PDMS), which is the optimal composition, the peak stress increases by 12.0%, 73.8%, and 66.9% over NS_0.5_-CHM_3.5_/PDMS at strain rates of 2500 s^−1^, 3500 s^−1^, and 4500 s^−1^, respectively. Suitable introduction of NS significantly enhances the strength of the as-prepared composite, which could be attributed to the high rigidity of NS, strengthening the structural strength of the PDMS matrix. Notably, the strength enhancement observed at the highest strain rate (4500 s^−1^) is lower than that at 3500 s^−1^, which can be attributed to local softening of the PDMS matrix induced by temperature rise under excessively high strain-rate loading. When the NS content exceeds 1 wt.%, a decrease in composite strength is observed, which results from the formation of agglomerates through van der Waals forces among the high-specific-surface-area nanoparticles. The agglomerates exhibit weak interfacial bonding with the matrix, impairing efficient stress transfer and acting as sites for crack initiation, thereby exacerbating stress concentrations and ultimately weakening the material strength. The compressive modulus (Figure 5c) and energy absorption per unit volume (Figure 5e), which were obtained through an integration of the stress–strain curve, initially increase and then decrease with NS content, reaching the peak values at 1 wt.% NS content, same as the trend in the compressive strength. The micro-scale CHMs absorb energy through cavity collapse, thereby delaying the failure of the composite material. Simultaneously, the nano-scale NS particles form a rigid network that enhances the strength of the PDMS matrix and impedes crack propagation. Together, CHM and NS collaborate to achieve a multi-level structural energy dissipation design across different scales.

Considering their weight for lightweight impact protection, the specific mechanical performance of the composites was further studied, which was calculated by the division of corresponding mechanical properties (i.e., strength, modulus, and energy absorption) over the density of samples, as depicted in Figure 5b,d,f. Despite the greater density of NS compared to CHM, its rigidity enables the NS-CHM/PDMS composites to balance the lightweight design with excellent energy absorption. The NS_01_-CHM_03_/PDMS exhibits superior mechanical performance under high-speed impact, resulting from the synergistic effect between the two fillers. As the hollow structure of CHM reduces the density and delays the structural failure, and the NS mitigates the negative impact of increased density through strengthening the PDMS matrix, the composites ultimately achieve a well-balanced overall performance.

**Figure 6:**
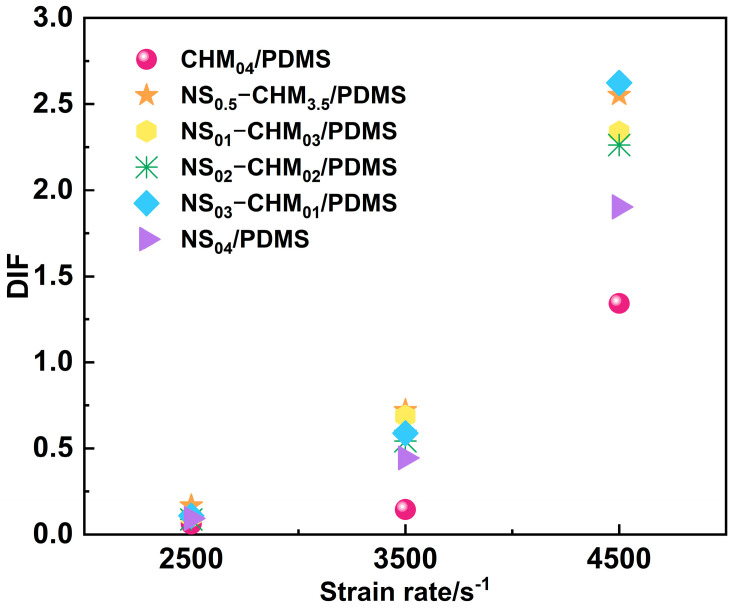
Relationship between dynamic increase factor (DIF) and strain rate for NS-CHM/PDMS composites.

Figure 6 quantitatively depicts the dynamic increase factor (DIF), defined as the ratio of dynamic strength to quasi-static strength, to systematically reveal the effect of NS/CHM introduction on the strain-rate sensitivity of PDMS composites. The results showed that the DIFs of all composites increase significantly with the strain rate, indicating an enhancement in their bearing capacity under high-speed impact. Strikingly, the NS_01_-CHM_03_/PDMS composite exhibits a DIF of merely 0.10 at 2500 s^−1^, which surges to 2.33 at 4500 s^−1^ with a 2273% increase. The dramatic increase vividly reflects the strengthening effect of the NS/CHM on the dynamic mechanical behavior of the composite. For the CHM_04_/PDMS composite, the DIF value is lower, which is attributed to the inherent strength of CHM being lower than that of NS. The improvement in its dynamic strength primarily relies on the densification effect caused by CHM collapse, but is limited by the structural strength of the PDMS matrix. For the NS_04_/PDMS composite, nanoparticle agglomeration caused by the excessive NS induction can lead to localized stress concentrations. Then, the crack propagates rapidly in the absence of CHM, leading to brittle fracture and early failure, which manifests as a low DIF. Both CHM_04_/PDMS and NS_04_/PDMS exhibit lower DIFs, further indicating that the dynamic impact performance of the composites could be effectively enhanced by the synergistic effect of CHM and NS.

**Figure 7:**
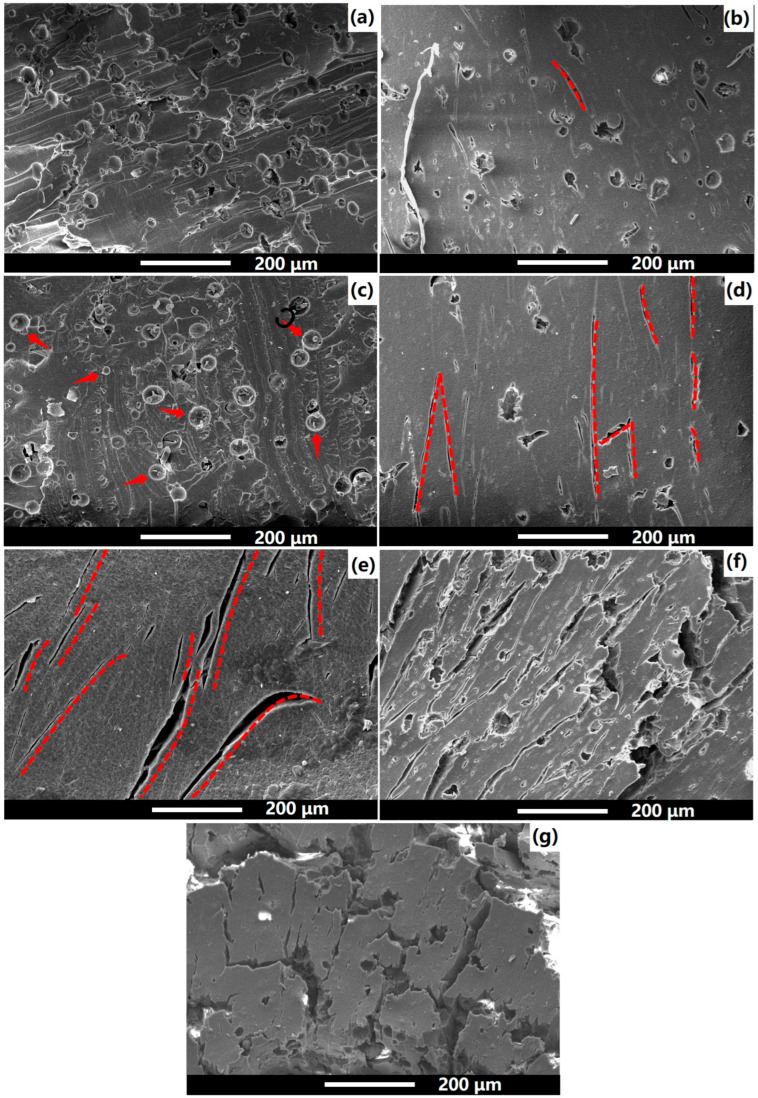
SEM images for the composite after high strain-rate impact: (**a**) NS_0.5_-CHM_3.5_/PDMS; (**b**) NS_01_-CHM_03_/PDMS; (**c**) NS_02_-CHM_02_/PDMS; (**d**) NS_03_-CHM_01_/PDMS; (**e**) NS_04_/PDMS at the strain rate of 2500 s^−1^. NS_01_-CHM_03_/PDMS at the strain rate of (**f**) 3500 s^−1^ and (**g**) 4500 s^−1^.

The fracture morphology after dynamic impact was studied to further understand the dynamic failure mechanism of NS-CHM/PDMS composites, as shown in Figure 7. As reported in our previous research [18], the energy absorption of CHMs primarily originated from the plastic collapse, manifesting as complex crack branching, which can significantly enhance the energy dissipation efficiency.

As CHM content decreases and NS content increases (Figure 7b,d,e), the crack propagation mode gradually changes from a complex branching mode to a linear propagation mode. For the NS_01_-CHM_03_/PDMS composite, which shows the best dynamic impact performance, the debonding between the CHM filler and PDMS matrix is less commonly observed, verifying that the introduction of a suitable amount of NS can build a rigid network for energy dissipation and reduce the amount of stress the CHMs have to bear [29]. Images for the NS_01_-CHM_03_/PDMS at varied strain rates (Figure 7b,f,g) further reveal that as the strain rate increases from 2500 s^−1^ to 4500 s^−1^, crack propagation accelerates in the PDMS matrix. In addition, the dominant failure behavior of CHM shifts from plastic collapse to brittle fracture. Under the highest strain rate of 4500 s^−1^, extensive interfacial debonding occurs, leading to a significantly shortened time for structural failure. The thermal stability of the composites can help understand the multifunctional properties of the as-prepared materials, which are detailed in the Supplementary Materials.

## 4. Conclusions

This study constructed NS and CHM co-reinforced PDMS composites and studied the mechanical behavior and energy dissipation mechanisms of the composites across a broad strain-rate range (2.8 × 10^−3^–4500 s^−1^). It is suggested that NS effectively fills the CHM/PDMS interfacial micro-gaps in composites and significantly enhances the interfacial bonding strength through Si–O–Si covalent bonding. At 1 wt.% NS loading (NS_01_-CHM_03_/PDMS), the compressive strength reaches 39.73 MPa—a 67% improvement with respect to the pure PDMS. Under dynamic impact (4500 s^−1^), the synergistic effect of the physical barrier effect from NS and the hollow-structure collapse of CHMs elevates energy absorption density to 40.18 J/g, representing an 179% enhancement over the CHM/PDMS composites with the same filler weight. The dynamic impact factor (DIF) of NS_01_-CHM_03_/PDMS surges by 2273%, confirming substantially optimized strain-rate sensitivity. Overall, the present study proposed a novel method for fabricating lightweight impact-resistant materials through the multi-scale synergistic effect.

## Figures and Tables

**Table 1:** Basic physical parameters of the preformed phenoset microspheres.

	Product Model	Bulk Density (g/cm^3^)	Average Compressive Strength (psi)	Moisture Content
Phenoset microspheres	BJO-0930	0.104	3	<4 wt.%

**Table 2:** Compositions of NS-CHM/PDMS composites.

NS (wt.%)	CHM (wt.%)	Sample Denotations
0	4	CHM_04_/PDMS
0.5	3.5	NS_0.5_-CHM_3.5_/PDMS
1	3	NS_01_-CHM_03_/PDMS
2	2	NS_02_-CHM_02_/PDMS
3	1	NS_03_-CHM_01_/PDMS
4	0	NS_04_/PDMS

## Data Availability

The original contributions presented in this study are included in the article and Supplementary Materials. Further inquiries can be directed to the corresponding authors.

## Supplementary Materials

The following supporting information can be downloaded at: https://www.mdpi.com/article/10.3390/polym17192592/s1, Figure S1: Quasi-static stress-strain curves of the NS/CHM-PDMS composites with varied filler composition; Figure S2: Quasi-static stress-strain curves of Pure PDMS; Figure S3. Thermogravimetric curves of NS-CHM/PDMS composites; Figure S4 DSC curve for the NS04/PDMS composite; References [30,31] are cited in the supplementary materials.
