# Supplementary material for: Mechanisms Underpinning Dynamic Impact Resistance Reinforcement by Multi-Scale Synergistic Effect Through Nano-Silica and Carbon Hollow Microsphere
Source: Polymers (Basel). 2025 Sep 25;17(19):2592. doi: 10.3390/polym17192592 (PMC12526741; doi:10.3390/polym17192592)
Supplement: Supplementary file 1 [file polymers-17-02592-s001.zip › polymers-3867044-SI.pdf]

Supplementary information for

Mechanisms Underpinning Dynamic Impact Resistance Reinforcement by Multi-Scale Synergistic Effect Through Nano-silica and Carbon Hollow Microsphere

Yingying Yu, Cheng Yang, Yaxi Zhang, Linjia Wang, Hong Wang, Fandong Meng, Fanyi Meng *, Tao Wang and Zhenmin Luo *

College of Safety Science and Engineering, Xi'an University of Science and Technology, Xi'an 710054, China; yyyu2022@xust.edu.cn (Y.Y.); cyangxust2024@163.com (C.Y.); komoriyui_1152@outlook.com (Y.Z.); wanglinjia@stu.xust.edu.cn (L.W.); 17308387551@163.com (H.W.); mengf@xust.edu.cn (F.M.); christfer@xust.edu.cn (T.W.)

* Correspondence: mengfy836@foxmail.com (F.M.); zmluo@xust.edu.cn (Z.L.)

Figures:

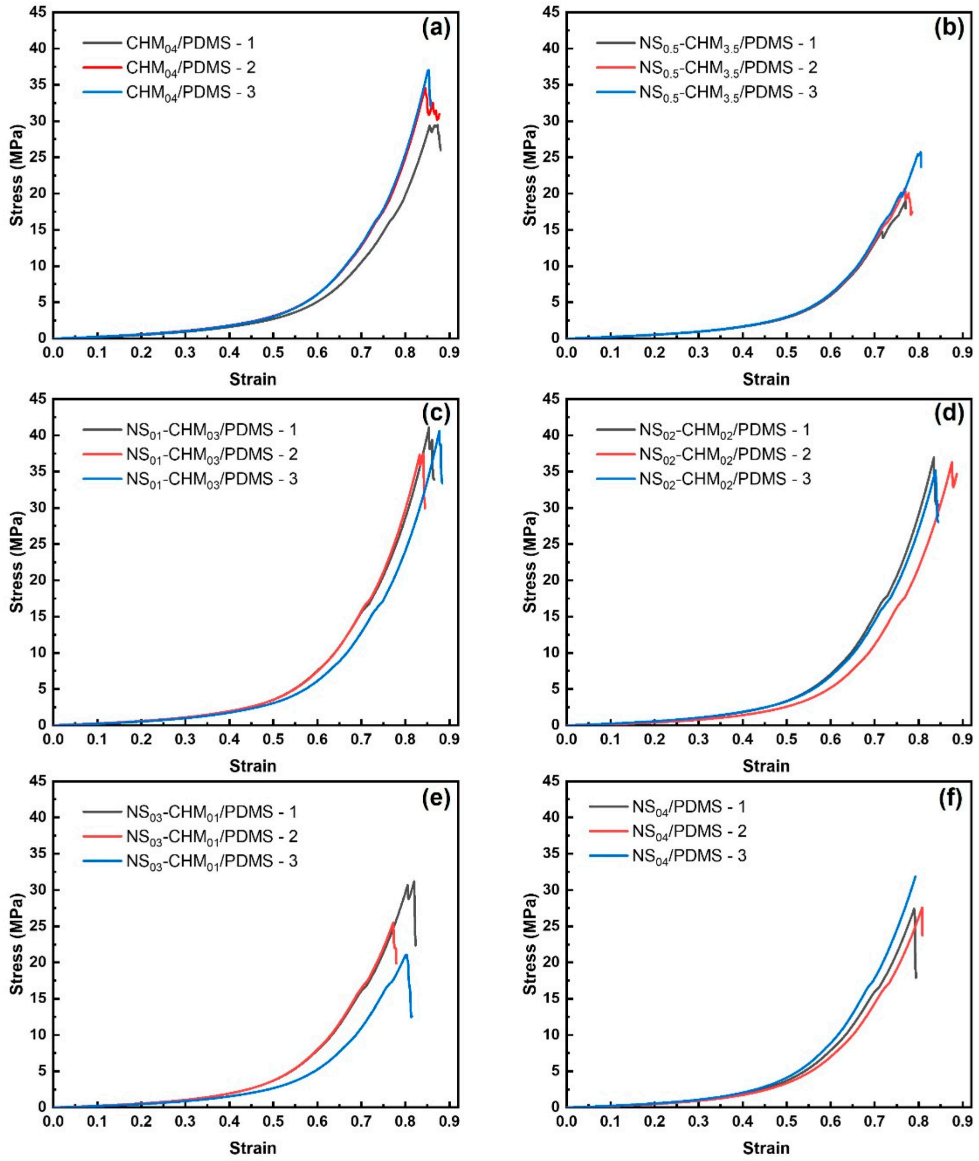

Figure S1 Quasi-static stress–strain curves of the NS/CHM-PDMS composites with varied filler composition.

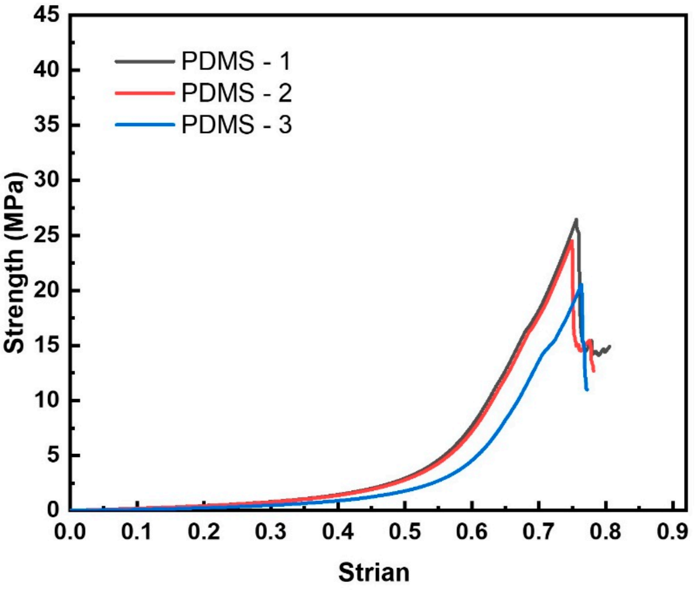

Figure S2 Quasi-static stress–strain curves of pure PDMS.

Thermal Stability of NS-CHM/PDMS Composites

Thermogravimetric analysis was conducted to study the thermal stability of the composites, as shown in **Figure S3**. A slight increase can be observed at the beginning, which might be attributed to the further curing of the PDMS matrix. The main weight loss of the thermal decomposition process occurred within the temperature range of 300–450°C. During this stage, the PDMS backbone undergoes significant thermal cleavage, generating gases such as methane and cyclic siloxanes, while simultaneously converting into a silica residue [1-2]. The residue components after the TGA could be dual-phase solid components consisting of silica and carbon. With the increased NS content, the residue of NS-CHM/PDMS composites gradually increased, and NS_{03}-CHM_{01}/PDMS exhibited the highest residue rate of 61.70%. Meanwhile, the initial decomposition temperature also enhanced with the increase in NS content, proving that the introduction of NS can effectively improve the thermal stability of the as-prepared composites.

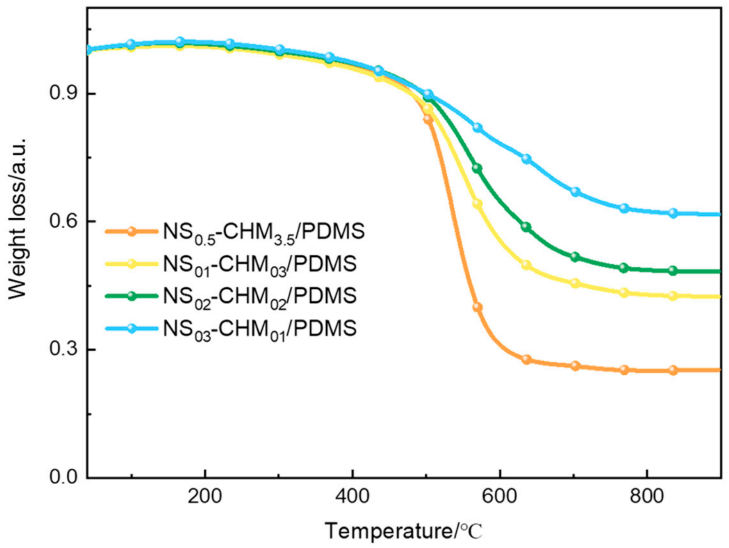

Figure S3. Thermogravimetric curves of NS-CHM/PDMS composites.

The temperature of maximum weight loss rate enhanced with increased NS content, peaking at 572.48 °C for the NS_{03}-CHM_{01}/PDMS composite, confirming that the nano-micro hybrid structure synergistically delayed the thermal decomposition of the as-prepared composite. Further improving the NS content, for the composite with 4wt.% NS, the agglomeration of the nanoparticles can act as the heat concentration point, which is not favorable for enhancing the thermal stability.

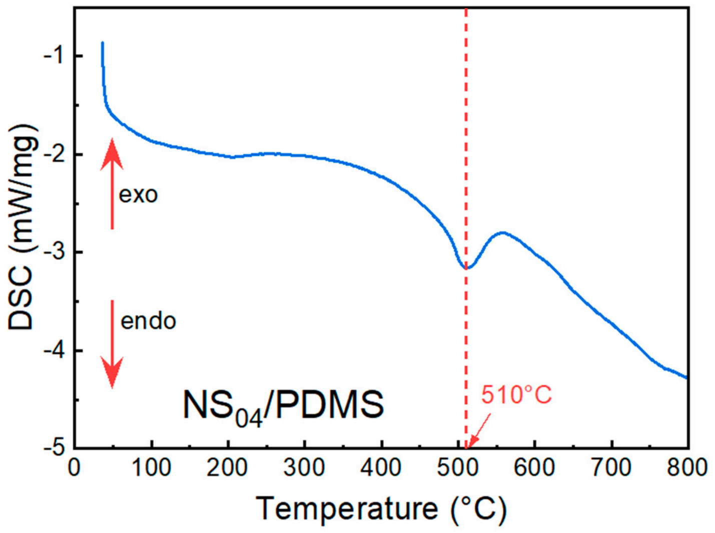

Figure S4 DSC curve for the NS_{04}/PDMS composite.

References

[1] Wang, J., Li, G., Zhang, Z., et. al. Detailed insights of polydimethylsiloxane (PDMS) degradation mechanism via ReaxFF MD and experiments. Chemical Engineering Journal, 2024, 488, 150728.

[2] Krishnan, G. S., Naveen, S., Shahnawaz, M., & Ramcharan, T. Pyrolysis and thermal degradation studies of silane-carbosilane transformation using hyphenated thermal analysis. Journal of Analytical and Applied Pyrolysis, 2022, *164*, 105535.
